# *Gossypium hirsutum* gene of unknown function Gohir.A02G161000 encodes a potential transmembrane Root UVB Sensitive 4 Protein with a putative protein-protein interaction interface

**DOI:** 10.17912/micropub.biology.000869

**Published:** 2024-03-01

**Authors:** Danielle Graffam, Marissa Cutlan, Amanda R Storm, Amanda M Hulse-Kemp, Angela K Stoeckman

**Affiliations:** 1 Chemistry Department, Bethel University, Saint Paul, MN USA; 2 Department of Biology, Western Carolina University, Cullowhee, NC USA; 3 Genomics and Bioinformatics Research Unit, The Agricultural Research Service of U.S. Department of Agriculture, Raleigh, NC USA; 4 Department of Crop and Soil Sciences, North Carolina State University, Raleigh, NC USA

## Abstract

A gene of unknown function, Gohir.A02G161000.1, identified in *Gossypium hirsutum* was studied using computational sequence and structure bioinformatics tools. The associated protein GhRUS4-A0A1U8JPV7 (UniProt A0A1U8JPV7) is predicted to be a plastid-localized, transmembrane root UVB-sensitive 4 (RUS4) protein with a newly identified potential dimerization surface. Evidence from homology and sequence conservation suggest involvement in auxin transport and pollen maturation.

**Figure 1. Sequence and Structure Characterization of GhRUS4-A0A1U8JPV7:**
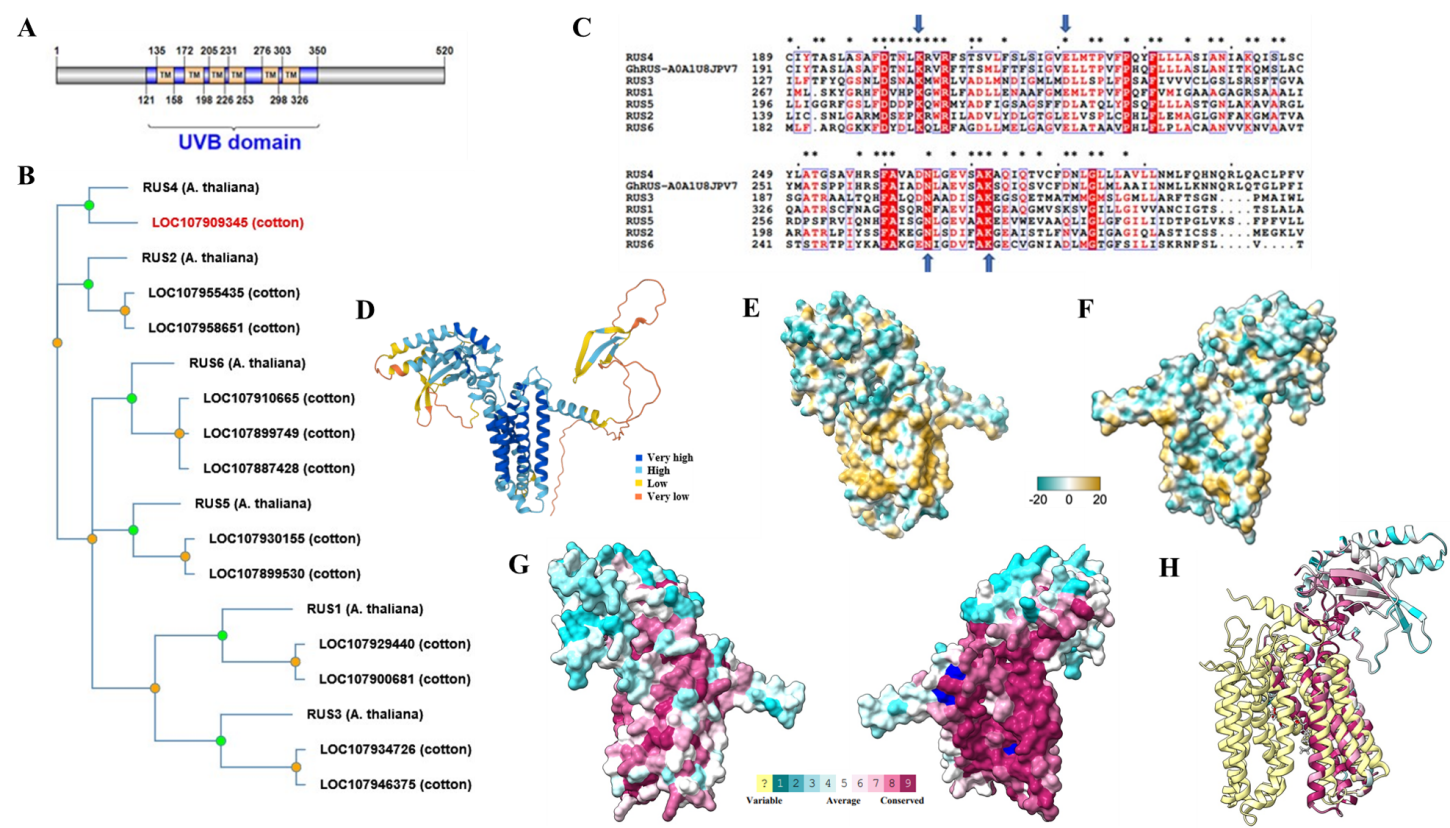
(A) Domain architecture of GhRUS4-A0A1U8JPV7 indicating the location of predicted sequence features, created using Illustrator for Biological Sequences (Liu et al. 2015) based on predictions from InterPro (Blum et al. 2020) and MemBrain 3.1 (Feng et al. 2020). TM - transmembrane domain, UVB domain - vitamin B6 photo-protection and homeostasis domain (PF04884). (B) Phylogenetic tree demonstrating the relationship of GhRUS4-A0A1U8JPV7 (highlighted in red) and other cotton paralogs to the six *Arabidopsis thaliana* root UVB sensitive (RUS) family members, created using PhyloGenes (Zhang et al. 2020). (C) Multi-Sequence Alignment of GhRUS4-A0A1U8JPV7 with Arabidopsis homolog across a section of the UVB domain, prepared with ClustalOmega (Madeira et al. 2019) and ESPript3 (Robert and Gouet 2014). ConSurf-identified (Ashkenazy et al. 2016) highly conserved sequences (*) and residues involved in AtRUS1/AtRUS2 dimerization (arrows) are highlighted (Leasure et al. 2009). (D) AlphaFold (Jumper et al. 2021) model structure of GhRUS4-A0A1U8JPV7 viewed in ChimeraX (Pettersen et al. 2021) with very high confidence predictions in dark blue. The disordered, low-confidence region at the N-terminus was removed from the structure in all subsequent analyses. (E) Hydrophobic surface coloration of GhRUS4-A0A1U8JPV7 model calculated by ChimeraX, (F) Structure rotated by 180 degrees. Blue indicates low hydrophobicity and brown indicates highly hydrophobic regions. (G) ConSurf identified conserved residues (dark purple) mapped to structure surface in same orientations as E and F, Dark blue residues highlight residues identified as important in AtRUS1/AtRUS2 dimerization shown in C (Leasure et al. 2009). (H) Overlay of GhRUS4-A0A1U8JPV7 (Consurf coloration) with structural homolog melibiose permease (PDB 7L16; light yellow), structure orientation rotated 90 degrees clockwise along the long axis relative to F.

## Description

Introduction

Cotton has been cultivated for at least 7,000 years, fueling the world as a fiber and food crop. From field to fabric, cotton is indispensable to the United States economy, accounting for more than $21 billion in products and services annually and producing all types of apparel from sheets to towels to tents (USDA ERS, 2022) . The allotetraploid cotton genome is relatively large but does retain genome stability across generations and geographic distributions (Chen ZJ et al. 2020) . This large genome required conceptual advances and epigenetic and genomic resources in order to sequence. Improvements in computational data enabled the discovery of reading frames as well as prediction of protein function, yielding a fully sequenced upland cotton ( *Gossypium hirsutum* L. accession Texas Marker-1 (TM-1) version 2.0) in 2020 with accompanying annotation version 2.1 (Chen ZJ et al. 2020) . Even after analysis, it was found that thousands of genes could not be assigned a function.

We present evidence that one gene of unknown function, LOC107909345 (Gohir.A02G161000.1_UTX-TM1_v2.1, CottonGen: https://www.cottongen.org/bio_data/5890879 ) and associated protein (NCBI: KAG4212371.1; UniProt: A0A1U8JPV7), here referred to as GhRUS4-A0A1U8JPV7, is part of the root ultraviolet B (UVB) sensitive (RUS) protein family. Most plant species have between five and sixteen RUS genes, while species within the animal kingdom typically have a single gene. *Arabidopsis* has six family members that appear to function in multiple stages of plant development, although it is still unclear if some are functionally redundant. Knockouts of AtRUS1 and AtRUS2 were arrested during germination in a UVB-dependent manner that could be partially rescued by high concentrations of vitamin B6 (Tong et al. 2008; Leasure et al. 2009) . AtRUS1 and AtRUS2 (also known as weak auxin response WXR3 and 1, respectively) were shown to physically interact and are implicated in auxin transport, responses to UV-B exposure, and seedling development (Leasure et al. 2009; Ge et al. 2010; Yu et al. 2013) . In addition, these RUS family members may be important to vitamin B6-dependent homeostasis in plants through interaction with aspartate aminotransferase 2 (Leasure et al. 2011) . Less is known about the remaining AtRUS members. When AtRUS3, AtRUS4, or AtRUS5 knockouts were grown under standard conditions, no noticeable morphological differences from wild-type were observed (Perry et al. 2021) ; although knockdown of AtRUS4 disrupted anther dehiscence and pollen maturation, indicating a role in jasmonate mediated maturation of stamen and pollen (Chen YJ et al. 2020; Zhao et al. 2019) . AtRUS6 homozygous knockouts were found to be embryonic lethal at the transition to globular stage (Perry et al. 2021) .

Sequence Features

The InterPro web server (Blum et al. 2020) identified the 521-amino acid GhRUS4-A0A1U8JPV7 protein as a member of the root UVB sensitive (RUS) protein family (IPR006968), with a ‘Vitamin B6 photo-protection and homeostasis’ (DUF647) domain (PF04884). A domain architecture was created to visualize sequence features ( **Figure 1A** ). Transmembrane (TM) prediction was variable with predictions of 2 to 6 transmembrane helices depending on the program (HMMTOP, Tusnady and Simon 2001; Phobius, Käll et al. 2007; TMHMM, Hallgren et al. 2022; MemBrain 3.1, Feng et al. 2020). Previously, 0-4 transmembrane segments were variably predicted in the *Oryza sativa* (rice) RUS family (Yu et al. 2016) . Positioning of TM regions in the domain architecture were further supported by the structural model described below.

Sequence analysis of GhRUS4-A0A1U8JPV7 by subcellular localization programs YLoc (Briesemeister et al. 2010a; Briesemeister et al. 2010b), TargetP 2.0 (Armenteros et al. 2019), Localizer (Sperschneider et al. 2017) , and Plant-mSubP (Sahu et al. 2020) predicted the location to be in the plastid with medium to high confidence, and BUSCA (Savojardo et al. 2018) predicted an organelle membrane location with moderate confidence. This is in agreement with *Arabidopsis* RUS homologs 1, 2, 4 and 6 which have been experimentally localized to the plastid (Yu et al. 2016; Ge et al. 2010; Zhao et al. 2019; Perry et al. 2021) . Evidence supports the localization of *Arabidopsis* RUS1 and RUS2 to the membranes of plastids based on the bioinformatics of solute transporters and their evolutionary origins (Tyra et al. 2007) and proteomics of *Arabidopsis* chloroplast envelope membranes (Ferro et al. 2003) .

Homology

The genome of *Arabidopsis thaliana* contains six RUS family members ( *RUS1* AT3G45890; *RUS2* AT2G31190; *RUS3* AT1G13770; *RUS4* AT2G23470; *RUS5* AT5G01510; *RUS6* AT5G49820). A PhyloGenes (Zhang et al. 2020) phylogenetic tree based on gene families, including cotton and *Arabidopsis* homologs ( **Figure 1B** ), indicated that GhRUS4-A0A1U8JPV7 (red sequence) clustered with *Arabidopsis* RUS4 (58.2% identical over 95% of the query coverage in a BLASTp alignment). *Arabidopsis* RUS4 was experimentally shown to be located in the chloroplast and is most highly expressed in the mature leaf, with knockdown resulting in defective pollen maturation and reduced male fertility (Chen YJ et al. 2020; Zhao et al. 2019) . Interestingly, no RUS4 homolog exists in the rice genome, although it was found in gymnosperm and *Charophyte* algae genomes (Perry et al. 2021) .

ConSurf (Ashkenazy et al. 2016) was used to calculate the evolutionary conservation of each amino acid residue in GhRUS4-A0A1U8JPV7, many of which resided within the UVB domain. These highly conserved residues are indicated by asterisks in the multi-sequence alignment (MSA) of GhRUS4-A0A1U8JPV7 with the *Arabidopsis* homologs ( **Figure 1C** ). Some highly conserved residues are shared across all homologs, including the 4 residues implicated in the dimerization of AtRUS1 and AtRUS2 (arrows) (Leasure et al. 2009) . The full ConSurf results and ClustalOmega alignment for the MSA are available as Extended Data.

Structural Features

The AlphaFold (Jumper et al. 2021) structural model for GhRUS4-A0A1U8JPV7 showed the high-confidence folded regions were mostly alpha helical, with two anti-parallel beta strands near the C-terminus and a bundle of 6 helices in the middle of the polypeptide ( **Figure 1D** ). These helices match the transmembrane regions predicted by some programs such as MemBrain 3.1. A hydrophobic surface analysis shows that one face of this helix bundle is hydrophobic (dark gold in **Figure 1E** ) while the other face is largely hydrophilic (blue in **Figure 1F** ). This could explain why the aforementioned prediction programs were variable in their ability to predict some of these helices as transmembrane helices. ConSurf conservation coloring of the surface showed that the hydrophilic face was highly conserved ( **Figure** **1G** ). The large, conserved face suggests this may be a protein-protein interaction interface and that GhRUS4-A0A1U8JPV7 may function as a homo- or hetero-dimer.

It has been suggested that AtRUS1 and AtRUS2 interact in a DUF647 dependent-manner and form a complex necessary for function (Leasure et al. 2009) . Site-directed mutagenesis of AtRUS1 within that domain at K281G and K349G completely abolished interaction with AtRUS2 in a yeast two-hybrid assay, while E298G and N342G mutations severely weakened the interaction. When these four residues, which are conserved in GhRUS4-A0A1U8JPV7, are mapped onto the model structure, 3 of these residues (E223, N267, K274) are exposed on the conserved face of the potential protein-protein interaction interface ( **Figure 1G,** dark blue **)** which agrees with the experimental findings for AtRUS1 and AtRUS2.

A DALI search (Holm 2020) for structures similar to GhRUS4-A0A1U8JPV7 found the closest matches to be various transporter proteins such as melibiose permease (PDB 7L16; Z-score 14.7; rmsd 3.4). An overlay of the GhRUS4-A0A1U8JPV7 ConSurf-conservation model with the structure of the bacterial transporter melibiose permease ( **Figure 1H** ) demonstrates structural similarity between the 6-helix bundle of the transmembrane region and half of the melibiose permease. Thus, a dimer between two cotton RUS proteins could form a structure similar to the complete transporter. These structural features suggest an as-yet unreported feature of RUS proteins, that GhRUS4-A0A1U8JPV7 is an integral membrane protein that dimerizes through its transmembrane helical bundle to form a hydrophilic channel which could function as a transporter. The inclusion of Arabidopsis RUS proteins in a list of plastid solute transporters (Tyra et al. 2007) supports this potential transporter role.

Conclusion

Evidence from sequence analysis indicates GhRUS4-A0A1U8JPV7 is a transmembrane protein localized to the plastid membrane. Homology exploration identifies GhRUS4-A0A1U8JPV7 as a member of the RUS protein family with the closest homology to the Arabidopsis RUS4 subfamily. Structure modeling and residue conservation indicate that this protein could be serving as a membrane transporter by dimerization at the hydrophilic face of a transmembrane six-helix bundle.

## Extended Data

Description: ConSurf sequence conservation results. Resource Type: Dataset. DOI: 10.22002/13rs1-pnx26
